# Beyond Infection: How Antimicrobial Therapies Influence the Urinary Microbiome and Stone Disease

**DOI:** 10.3390/ph18071038

**Published:** 2025-07-12

**Authors:** Oana Nicu-Canareica, Vlad-Octavian Bolocan, Loredana Sabina Cornelia Manolescu, Petru Armean, Cosmin Medar, Liliana Burlibașa, Maria-Luiza Băean, Viorel Jinga

**Affiliations:** 1 Doctoral Program Studies, University of Medicine and Pharmacy “Carol Davila”, 050474 Bucharest, Romania; oana.canareica@drd.umfcd.ro (O.N.-C.); vlad-octavian.bolocan@drd.umfcd.ro (V.-O.B.); 2 Department of Fundamental Sciences, Faculty of Midwifery and Nursing, University of Medicine and Pharmacy “Carol Davila”, 050474 Bucharest, Romania; loredana.manolescu@umfcd.ro (L.S.C.M.); ml_baean@yahoo.com (M.-L.B.); 3 Department of Clinical Laboratory of Radiology and Medical Imaging, Clinical Hospital “Prof. Dr. Theodor Burghele”, 050664 Bucharest, Romania; 4 Clinical Laboratory of Medical Microbiology, Marius Nasta Institute of Pneumology, 050159 Bucharest, Romania; 5 Discipline of Public Health and Management, Department of Specific Sciences, Faculty of Midwifery and Nursing, University of Medicine and Pharmacy “Carol Davila”, 050474 Bucharest, Romania; 6 Genetics Department, Faculty of Biology, University of Bucharest, 060101 Bucharest, Romania; liliana.burlibasa@bio.unibuc.ro; 7 Department of Urology, Clinical Hospital “Prof. Dr. Theodor Burghele”, Faculty of Medicine, University of Medicine and Pharmacy “Carol Davila”, 050474 Bucharest, Romania; viorel.jinga@umfcd.ro; 8 Department of Urology, Clinical Hospital “Prof. Dr. Theodor Burghele”, 050664 Bucharest, Romania; 9 Medical Sciences Section, Academy of Romanian Scientists, 050085 Bucharest, Romania

**Keywords:** urolithiasis, microbiota, antibiotics, dysbiosis, infection, probiotics

## Abstract

The discovery of a resident urinary microbiome has significantly altered the understanding of urolithiasis, expanding its etiology beyond metabolic and dietary factors to include microbial contributions. This review highlights how specific uropathogens—particularly *Proteus mirabilis*, *Klebsiella pneumoniae*, and *Escherichia coli*—facilitate stone formation through mechanisms such as urease activity, citrate degradation, urine alkalinization, biofilm development, and inflammatory signaling. We critically examine how antibiotic therapies, while essential for treating urinary tract infections (UTIs), disrupt microbial homeostasis by depleting beneficial taxa like *Lactobacillus* and enabling colonization by lithogenic and resistant strains. Recurrent or broad-spectrum antibiotic use is linked to persistent dysbiosis and increased risk of stone recurrence. Additionally, this paper explores emerging microbiome-targeted strategies—such as probiotics, prebiotics, bacteriotherapy, and precision antimicrobials—as potential interventions to restore microbial balance and mitigate stone risk. Recognizing the urinary microbiome as a therapeutic target opens new avenues for personalized, microbiota-conscious strategies in the prevention and management of kidney stone disease.

## 1. Introduction

For decades, the urinary tract was considered a sterile environment, free of microbial life in healthy individuals. This dogma persisted primarily due to the limitations of traditional urine culture techniques. However, recent advances in next-generation sequencing and metagenomic analysis have dramatically changed this view, revealing a complex urinary microbiome that plays a vital role in human health and disease. This paradigm shift has been particularly impactful in the study of urolithiasis—commonly known as kidney stone disease—where microbial factors are now seen as potential contributors not only to stone genesis but also to recurrence and disease progression [1,2].

Kidney stones represent a major global public health issue due to their increasing prevalence, high recurrence rate, and significant healthcare costs. While metabolic and dietary factors have historically been emphasized, emerging data indicate that microbial communities residing in the urinary tract may contribute to stone formation. For example, species such as *Proteus mirabilis*—known for its urease activity—and *Oxalobacter formigenes*—an oxalate-degrading bacterium—have been identified as key microbial actors in either promoting or mitigating lithogenesis, depending on the context of microbial balance and host interactions [3,4].

Moreover, the intricate crosstalk between the urinary microbiome, host metabolic pathways, and immune surveillance mechanisms constitutes a multifactorial network that extends beyond lithogenesis to encompass broader urological and systemic pathophysiological states. Biofilm-forming bacteria such as *Proteus mirabilis* and *Klebsiella pneumoniae* can serve as nucleation sites by anchoring mineral crystals within their extracellular polymeric matrix, thereby facilitating stone maturation and persistence. Concurrently, microbial metabolites—including ammonia, oxalate, and lipopolysaccharides—modulate urinary pH, oxidative stress, and epithelial barrier integrity, all of which are critical determinants of crystal aggregation, retention, and inflammation-mediated tissue remodeling [5,6].

Emerging evidence suggests that regional and population-specific factors—including dietary habits, antibiotic usage patterns, and healthcare infrastructure—can significantly influence urinary microbiome composition and, consequently, urolithiasis susceptibility. In countries such as Romania, where dietary patterns and antibiotic exposure differ from Western standards, these factors may contribute to distinct microbial profiles relevant to stone formation [7].

This review aims to consolidate the growing body of evidence linking the urinary microbiome to urolithiasis and to explore how antimicrobial therapies—long considered a solution for urinary tract infections—may inadvertently disrupt microbial ecosystems, leading to unintended consequences such as stone formation or disease recurrence. We highlight emerging diagnostic tools, therapeutic strategies, and interdisciplinary approaches that may redefine how urinary stone disease is understood and managed in the era of microbiome-informed medicine [8,9,10,11,12,13].

## 2. The Normal Bacterial Flora of the Urinary Tract

The urobiome, or urinary microbiota, refers to the community of microorganisms that inhabit the human urinary tract, typically existing as biofilms on the urothelial lining. Traditionally, urine was considered sterile in the absence of overt infection; this perspective was grounded in the limitations of standard urine culture methods [14,15]. However, the advent of culture-independent approaches, particularly 16S rRNA gene sequencing and metagenomics, has revealed that the urinary tract harbors a diverse microbial community even in healthy, asymptomatic individuals [16,17].

The composition of the urobiome is influenced by multiple factors, including sex, age, method of urine collection, hormonal status, and underlying health conditions [18,19]. Despite being less diverse than the gut or skin microbiomes, the urinary microbiome demonstrates unique resilience and functionality adapted to the specific physiological characteristics of the urinary tract, such as variable pH, osmolality, and flow dynamics, which restrict colonization by many bacterial taxa [20,21].

Predominant genera consistently identified in healthy individuals include *Lactobacillus*, *Corynebacterium*, *Streptococcus*, *Prevotella*, *Gardnerella*, *Staphylococcus*, and *Aerococcus*. These microbes are believed to contribute to urinary tract homeostasis, potentially through colonization resistance against uropathogens or modulation of local immune responses [22,23]. Sex-specific differences in urobiome profiles have been repeatedly observed, with women showing higher abundances of *Lactobacillus*, *Gardnerella*, and *Streptococcus*, a pattern likely shaped by anatomical differences and estrogen-mediated immune and microbial regulation [24,25].

Age is another crucial determinant of the urobiome. Shifts in microbial communities have been documented across the lifespan, with significant alterations occurring during puberty, menopause, and advanced age [26]. Aging and health status—particularly in conditions such as urinary incontinence, overactive bladder, and pelvic floor dysfunction—have been linked with urobiome dysbiosis, suggesting a strong connection between microbial imbalance and urological disease risk [11,27].

Understanding the dynamics of the urobiome is essential not only for refining diagnostic tools but also for tailoring antimicrobial strategies that minimize collateral disruption of these delicate microbial communities. This highlights the importance of personalized, microbiome-conscious approaches in the management of urinary health.

## 3. Host Factors Influencing Urolithiasis Risk: Demographic, Clinical, and Lifestyle Interactions

Urolithiasis risk is influenced by a multifactorial set of host-related factors, including sex, age, comorbidities, drug exposure, and nutritional status. These variables not only influence stone composition and recurrence but also modulate the structure and function of the urinary microbiome—an increasingly recognized contributor to lithogenesis. Microbiome profiles also vary across populations, potentially contributing to regional differences in stone composition, recurrence, and clinical outcomes.

### 3.1. Population-Specific Microbiome Profiles

Emerging data indicate that urinary and gut microbiome compositions vary substantially across populations, influenced by regional dietary practices, antibiotic use, and healthcare settings. A meta-analysis found that study location—alongside factors like age and stone composition—accounted for significant variations in microbiome profiles among urolithiasis patients, with genera such as *Prevotella* showing divergent roles across cohorts [28].

Data from Eastern Europe remain underrepresented. For example, a multicenter retrospective study identified *Escherichia coli* as the predominant uropathogen, followed by *Klebsiella* and *Proteus* spp., with fosfomycin maintaining high efficacy across all regions of Romania [29]. These findings suggest a relatively uniform pathogen profile but do not explore underlying microbiome differences.

An Eastern European study reported depletion of *Lactobacillus* and enrichment of *Enterobacteriaceae* among stone formers [29]. However, other researchers observed that when diet and antibiotic exposure were controlled, regional microbiome differences diminished—suggesting that lifestyle factors may play a more pivotal role than geography [30,31].

Notably, the utility of *Lactobacillus* as a protective taxon is contested; other studies found that in certain Asian cohorts, *Lactobacillus* was unexpectedly abundant in stone formers, highlighting strain-level and ecological context-dependent effects [6].

In summary, microbial signatures in urolithiasis appear region-specific and subject to confounders—diet, antibiotic policies, and healthcare practices. To translate this into personalized prevention, longitudinal, multicenter studies are needed that integrate detailed metadata (diet, antibiotic history) with functional and strain-level metagenomics across Eastern European and other populations.

### 3.2. Sex Variable

Sex-related differences in urolithiasis have long been recognized, with epidemiological data traditionally showing a higher incidence in men, largely attributed to greater urinary excretion of calcium, oxalate, and uric acid, as well as increased prevalence of calcium oxalate stones [32]. However, more recent studies indicate a progressive narrowing of this gender gap, particularly in industrialized nations, which some authors attribute to changing dietary patterns, increasing obesity rates in women, and a convergence of lifestyle risk factors between sexes [24,25].

Hormonal influences, particularly estrogen, appear to play a pivotal role in modulating stone risk. Estrogen has been associated with increased urinary citrate excretion and enhanced microbial diversity, both of which exert inhibitory effects on calcium oxalate crystallization [33]. This may partially explain the lower stone incidence observed in premenopausal women. However, this protective mechanism diminishes post-menopause, potentially equalizing stone risk with age [32,34].

Despite these findings, the literature remains divided. Some studies have failed to identify a significant correlation between estrogen levels and stone prevention, while others report no consistent sex-based differences in urinary composition once dietary intake and comorbidities are controlled [24,25]. Furthermore, sex-specific differences in the urinary microbiome—such as increased Lactobacillus prevalence in females—have been proposed as an additional modulator of lithogenic potential, although evidence here is also variable and often limited by small cohort sizes or lack of longitudinal data [35,36].

A comparative overview of sex-related differences in stone risk due to hormonal influence and microbiota modulation is presented in Table 1.

### 3.3. Age

Elderly individuals are more susceptible to urolithiasis due to age-related changes in renal function, polypharmacy, dehydration, and reduced mobility [37,38]. Importantly, aging is associated with reduced microbial diversity in the urinary tract, increased incidence of asymptomatic bacteriuria, and greater vulnerability to antibiotic-induced dysbiosis—all of which may elevate stone risk [39,40]. However, some studies argue that stone recurrence decreases with age, raising the possibility that host–microbiome interactions may fundamentally differ in older populations [41].

### 3.4. Comorbidities and Infection

Comorbid conditions such as diabetes, chronic kidney disease, obesity, and recurrent urinary tract infections (UTIs) significantly elevate the risk of stone formation [42]. Recurrent UTIs caused by urease-producing bacteria like *Proteus mirabilis* and *Klebsiella pneumoniae* have been linked to struvite and apatite stone formation [2]. Meanwhile, systemic inflammatory diseases, respiratory infections, and malnutrition may indirectly contribute to stone risk by altering immune responses and nutritional metabolism, affecting oxalate and citrate handling [43,44,45]. Conflicting findings persist, with some studies downplaying the direct role of infections in non-struvite stones, highlighting instead metabolic and immune dysregulation as central drivers [46,47].

### 3.5. Alimentation and Nutritional Status

Diet is a modifiable but complex factor in stone pathogenesis. High intake of sodium, animal protein, and oxalate-rich foods has been associated with calcium oxalate stone formation, while inadequate fluid intake is a universally acknowledged risk factor [46,48,49]. Conversely, diets high in fruits, vegetables, and calcium (from dietary sources) appear protective. Malnutrition, particularly protein–energy deficiency, can disrupt urinary citrate levels, compromise mucosal immunity, and increase susceptibility to infections that foster stone development [41]. Dietary habits, often shaped from early life by familial and social influences, play a critical role in modulating gut and urinary microbiota, with downstream effects on metabolic pathways implicated in stone formation [50].

Obesity has also emerged as a significant risk factor, not only due to its metabolic effects but also through its association with altered urinary composition and increased incidence of renal infections. Obese individuals frequently present with low urinary pH, hyperoxaluria, and uric acid supersaturation—conditions favorable for lithogenesis [42,51]. Moreover, obesity is associated with low-grade chronic inflammation and hormonal dysregulation that can impair host immune defenses and mucosal barriers, promoting colonization by uropathogens. Studies have demonstrated that obesity is linked to shifts in both gut and urinary microbiota, often resulting in reduced microbial diversity and increased abundance of pro-inflammatory or urease-producing species, which may potentiate infection and stone recurrence [52,53]. These mechanisms are summarized in Table 2, highlighting the multifaceted impact of obesity on renal pathophysiology and microbial dysregulation.

However, the impact of specific dietary components remains controversial, with some meta-analyses showing inconsistent associations between oxalate-rich foods and stone incidence, likely due to differences in gut microbiota composition and oxalate metabolism [54,55].

### 3.6. Drug Exposure

Multiple classes of medications have been implicated in the pathogenesis of urolithiasis through pharmacologically distinct mechanisms. Loop diuretics promote hypercalciuria by increasing renal calcium excretion, while carbonic anhydrase inhibitors like topiramate induce hypocitraturia and alkaline urine, predisposing to calcium phosphate stone formation [56]. Protease inhibitors—commonly used in antiretroviral therapy—are associated with crystalluria and may directly precipitate in the renal collecting system [57]. Other drugs, such as triamterene, sulfonamides, and high-dose ascorbic acid, can contribute to stone formation through poorly soluble metabolites or alterations in urinary pH.

Emerging pharmacological agents, such as phytocannabinoids—cannabidiol (CBD) and tetrahydrocannabinol (THC)—have demonstrated antimicrobial and anti-inflammatory properties in vitro, with preliminary evidence suggesting modulatory effects on host microbiota and epithelial integrity. While promising, their direct relevance to stone formation remains unproven and warrants further clinical investigation [58,59].

## 4. 16S rRNA Sequencing in Urinary Microbiome Research

16S rRNA gene sequencing has emerged as a pivotal tool for exploring microbial communities, including those in human urine, challenging the long-held belief that urine is sterile. This method targets the conserved regions of the 16S rRNA gene found in all bacteria while leveraging variable regions to allow precise taxonomic classification [60,61,62]. In urinary microbiome research, 16S rRNA sequencing enables the detection of diverse bacterial communities, even in asymptomatic individuals with no signs of infection, offering significant insight into microbial ecology and health implications [63,64].

**Figure 1:**
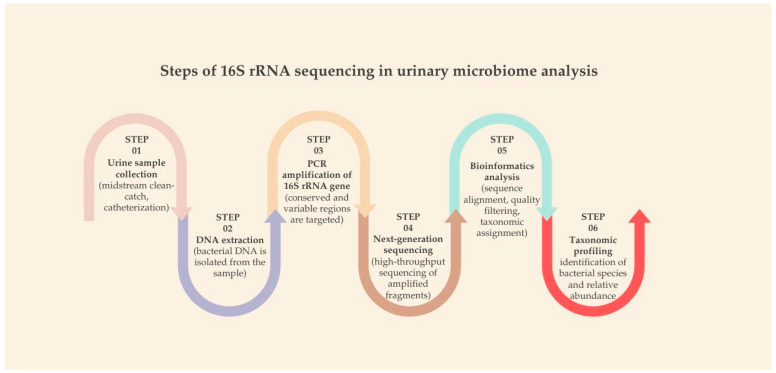
Steps of 16S rRNA sequencing in urinary microbiome analysis.

The standard workflow of 16S rRNA sequencing comprises several key steps: urine collection (via midstream, catheterization, or suprapubic aspiration), bacterial DNA extraction, PCR amplification of the 16S gene, high-throughput sequencing, followed by bioinformatics analysis and taxonomic profiling (Figure 1). This workflow allows researchers to map microbial diversity and abundance with relatively high resolution [65,66].

Compared to traditional culture-based diagnostics, 16S rRNA sequencing provides clear advantages. It is not dependent on bacterial viability or culture conditions, meaning it can detect fastidious or unculturable organisms. Moreover, it offers a broader overview of microbial communities, including low-abundance species that are often missed in conventional approaches. These attributes make it a powerful tool for studying the urinary microbiome, especially in low-biomass environments such as the bladder [19,67,68].

Nonetheless, limitations exist. The method lacks functional insight into microbial activity—it tells us “who is there,” but not necessarily “what they are doing.” Additionally, biases introduced during PCR amplification and sequencing platform limitations can affect taxonomic accuracy. Short-read sequencing often restricts classification to genus level, though careful selection of 16S regions (e.g., V1–V3 or V5–V8) and the use of curated databases can enhance species-level resolution [61,66,69].

Table 3 summarizes the main differences between 16S rRNA sequencing and conventional culture techniques. While 16S sequencing offers speed, sensitivity, and taxonomic breadth, it cannot fully replace culture in clinical microbiology, especially where antimicrobial susceptibility testing is required. An integrated diagnostic approach may ultimately provide the most robust clinical interpretation [6].

## 5. Bacterial Mechanism of Urinary Stone Formation

Bacteria contribute to urinary stone formation through multiple mechanisms, notably urease activity, alkalinization of urine, biofilm formation, citrate degradation, and inflammatory signaling [3,4,70]. Among these, the urease enzyme produced by *Proteus mirabilis* is particularly well-studied. Urease catalyzes the hydrolysis of urea into ammonia and carbon dioxide: Urea + H_2_O → 2 NH_3_ + CO_2_.

The resulting ammonia raises urine pH, promoting precipitation of calcium phosphate (hydroxyapatite) and magnesium ammonium phosphate (struvite), facilitating the formation of infection-induced stones [71].

Beyond urease activity, *Proteus mirabilis* and related species form biofilms on the urothelial surface or directly on the stone matrix. These EPS-protected microbial colonies are highly resistant to both antibiotics and host immune responses [71,72]. The biofilm matrix traps mineral ions and crystals, enhancing nucleation and aggregation, and promoting stone persistence [2,8,73].

Additional bacterial factors also facilitate stone pathogenesis. Citrate lyase, produced by some bacteria, degrades urinary citrate, a known inhibitor of calcium oxalate crystal growth. Reduced citrate levels predispose to calcium oxalate aggregation and stone formation. Moreover, urothelial injury caused by infection creates surfaces for crystal adhesion and nucleation [4,46].

Inflammatory processes also play a critical role. Uropathogens can induce pro-inflammatory cytokine production (e.g., IL-1β, IL-6, IL-8), altering urinary composition and promoting a pro-lithogenic microenvironment [74]. Chronic inflammation not only favors crystal retention but also enhances epithelial damage, completing a vicious cycle of infection and stone recurrence [8,75].

## 6. Specific Bacterial Species and Their Role in Lithogenesis

Certain bacterial species play central roles in urinary stone formation, particularly struvite and calcium oxalate stones (Table 4). Among them, *Proteus mirabilis*, *Klebsiella pneumoniae*, and *Escherichia coli* are the most frequently implicated pathogens [73,76,77].

### 6.1. Proteus *spp*.

1. Urease production: *Proteus mirabilis* is a potent urease producer. This enzymatic activity promotes the hydrolysis of urea into ammonia and CO_2_, creating an alkaline urine environment that favors struvite and apatite crystallization [3,78].
2. Urine alkalinization: Elevated pH from ammonia production precipitates magnesium ammonium phosphate (struvite) and calcium phosphate, both of which are key components of infection-induced stones [79].
3. Biofilm formation: *Proteus mirabilis* forms crystalline biofilms that contribute to persistent infection and serve as a nidus for mineral aggregation. These biofilms are highly resistant to both host defenses and antibiotics [70].

### 6.2. Klebsiella *spp.*

1. Urease production: *Klebsiella pneumoniae* also produces urease, albeit at lower levels than *Proteus mirabilis*, contributing to urine alkalinization and subsequent struvite formation [73].
2. Biofilm formation: *Klebsiella pneumoniae* forms robust biofilms, particularly in catheterized environments, often in synergy with other uropathogens [74,78].
3. Lithogenic effects: Experimental models have shown that *Klebsiella pneumoniae* enhances calcium oxalate (CaOx) crystal aggregation, acting as a nucleation site and promoting stone growth [80,81].

### 6.3. Escherichia coli

1. Lithogenic effects: Though non-urease producing, *Escherichia coli* is frequently isolated from calcium oxalate and struvite stones, suggesting alternative pathogenic mechanisms [2,55].
2. Citrate degradation: *Escherichia coli* expresses citrate lyase, which lowers urinary citrate—a key inhibitor of CaOx stone formation—thereby promoting lithogenesis [37].
3. Crystal growth: In vitro studies confirm *Escherichia coli*’s ability to increase both the number and size of CaOx crystals in a dose-dependent manner [2,55].
4. Biofilm formation: Similar to *Klebsiella*, *Escherichia coli* can establish persistent biofilms on urothelial surfaces or within catheter biofilms, further supporting infection–stone synergy [71,72].

Overall, these uropathogens contribute to stone pathogenesis via overlapping and synergistic mechanisms, including metabolic disruption, biofilm-mediated mineral retention, and inflammatory milieu alteration [30]. The frequent co-detection of these bacteria in both urine and stone matrices supports their etiological role in mixed-type urolithiasis [5].

## 7. Biofilm Formation on the Nucleus of a Urinary Stone

**Figure 2:**
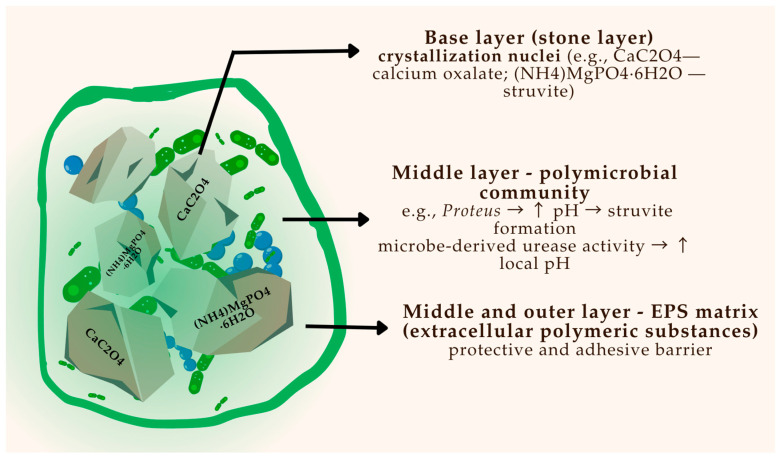
Schematic representation of a polymicrobial biofilm on the surface of a urinary stone.

The development of bacterial biofilms is a key contributor to stone initiation and persistence, particularly in infection-related calculi. Figure 2 provides a schematic overview of biofilm architecture on a urinary stone, illustrating microbial layering, EPS production, and bacterial entrapment.

Biofilm formation on the nucleus of a urinary stone is a structured, multistage process central to the pathogenesis of infection-related urolithiasis. The sequence begins with bacterial adhesion to epithelial or crystalline surfaces, followed by microcolony expansion and secretion of an extracellular polymeric substance (EPS) that stabilizes the microbial community [70,71].

**Figure 3:**
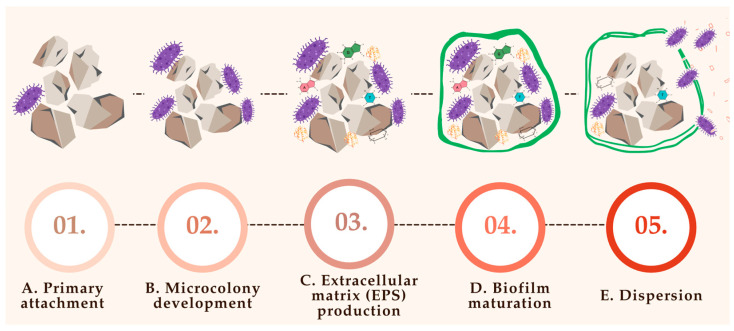
Biofilm formation on the nucleus of a urinary stone; (**A**)—primary attachment (bacteria adhere to stone surface via pili, adhesis, and proteins; organic film and urinary proteins enhance attachment); (**B**)—microcolony development (bacteria multiply and form early clusters controlled by urine pH, nutrient availability, and quorum sensing); (**C**)—extracellular matrix production (bacteria secrete extracellular polymeric substances (EPSs), which contain polysaccharides, proteins, and DNA that protect the biofilm); (**D**)—biofilm maturation (development of 3D architecture with nutrient channels); (**E**)—dispersion (bacteria detach due to stress (e.g., pH, nutrient depletion) leading to infection spread and new biofilm formation).

This matrix not only provides structural support but also facilitates mineral deposition, anchoring the biofilm to the stone surface and enhancing resistance to host defenses and antimicrobial agents [71]. Figure 3 provides a schematic representation of this process, illustrating the progressive stages of biofilm development and its integration with crystal nucleation and stone growth.

### 7.1. Primary Attachment (Figure 3A)

The initial adherence of bacteria to urinary stone surfaces represents a critical step in biofilm establishment and subsequent stone growth. This process, often termed primary attachment, involves bacterial structures such as fimbriae (pili), adhesins, and outer membrane surface proteins, which mediate tight binding to rough or porous stone surfaces [78].

Urinary stones typically possess an irregular surface topography enriched with organic residues derived from proteins, glycoproteins, and cellular debris, which facilitate microbial attachment [2]. Furthermore, urinary macromolecules—especially proteins excreted under inflammatory or infectious conditions—can form a conditioning film on the surface of stones, enhancing microbial anchorage and promoting colonization [23]. This early phase of microbial interaction with the stone substrate sets the stage for biofilm maturation and mineral entrapment, processes that are strongly associated with infection persistence and recurrent lithiasis [12].

### 7.2. Microcolony Development (Figure 3B)

Following initial adhesion, bacterial cells proliferate and organize into structured microcolonies on the surface of urinary stones. This phase marks a critical transition from single-cell attachment to early-stage biofilm formation, characterized by spatial organization and metabolic cooperation [78].

The development of microcolonies is influenced by several local environmental factors, including urine pH, the availability of nutrients, and the presence of competing or synergistic microbial species. Alkaline conditions—often induced by urease-producing bacteria such as *Proteus mirabilis*—further support biofilm maturation by enhancing matrix stability and facilitating mineral precipitation [82].

Importantly, bacteria within microcolonies engage in quorum sensing, a communication mechanism that enables coordinated gene expression in response to population density. This facilitates the regulation of key behaviors such as EPS production, biofilm expansion, and resistance mechanisms [83]. Through this cooperative behavior, microcolonies become highly resilient, promoting chronic infection and directly contributing to the persistence and enlargement of urinary stones [83].

### 7.3. Extracellular Matrix (EPS) Production (Figure 3C)

As bacterial microcolonies mature on urinary stone surfaces, they begin secreting extracellular polymeric substances (EPSs) that constitute the structural and functional core of the developing biofilm. The EPS matrix forms a cohesive and protective scaffold that embeds bacterial cells and anchors them to the substrate [71].

This heterogeneous matrix is primarily composed of polysaccharides, proteins, extracellular DNA (eDNA), and lipids, all of which contribute to the stability and resilience of the biofilm [71,84]. The EPS not only provides mechanical integrity to the biofilm but also creates a diffusion barrier that protects the bacterial community from antimicrobial agents and immune cell infiltration [51].

In the context of urolithiasis, EPS promotes mineral entrapment and crystal aggregation, playing a direct role in the expansion and persistence of urinary stones. Its hydrophobic and adhesive properties facilitate binding to both epithelial surfaces and crystalline material, linking infection to stone pathogenesis in a self-sustaining cycle [85].

### 7.4. Biofilm Maturation (Figure 3D)

As development progresses, the biofilm evolves into a complex, three-dimensional structure characterized by architectural features such as water channels, which allow for efficient nutrient diffusion and waste removal [72]. This stage marks the transition from an early colonization phase to a stable microbial consortium capable of long-term persistence on urinary stone surfaces.

Within mature biofilms, bacteria exist in heterogeneous physiological states, ranging from metabolically active to dormant or persister cells, which enhances community survival under hostile conditions such as antimicrobial therapy or host immune attack. This stratified organization enables the population to withstand environmental stress and contributes to chronic infection and treatment failure [78].

Furthermore, biofilm complexity often increases as new bacterial species colonize the established matrix, creating multi-species communities with synergistic interactions. These interactions can lead to enhanced virulence, broader metabolic capacity, and increased stone-promoting potential [70].

### 7.5. Dispersion (Figure 3E)

In the final stage of biofilm development, a subset of bacterial cells may undergo detachment from the mature biofilm, a process known as dispersion. This event enables bacteria to colonize new surfaces within the urinary tract or migrate to distant anatomical sites, potentially initiating secondary infections or new foci of stone formation [72].

Dispersion can be triggered by a variety of environmental cues, including nutrient limitation, accumulation of metabolic by-products, pH fluctuations, or the action of quorum-sensing molecules [8,71]. These signals activate genetic pathways that promote biofilm thinning, enzymatic degradation of the EPS matrix, and increased motility of planktonic cells.

Dispersed bacteria retain increased resistance traits acquired during their time in the biofilm, including antibiotic tolerance and immune evasion, making subsequent infections particularly challenging to eradicate. Their ability to reattach and establish new biofilms plays a crucial role in the chronicity and recurrence of urolithiasis associated with infection [2,86].

Different bacterial species contribute to urinary stone formation through distinct, yet sometimes overlapping, biochemical and structural mechanisms:

*Proteus* spp., particularly *Proteus mirabilis*, play a central role in biofilm-associated urolithiasis through urease production, which alkalinizes the urine, thereby promoting precipitation of phosphate salts (struvite and apatite) and facilitating crystal aggregation [76].

*Klebsiella* spp., including *Klebsiella pneumoniae*, are also capable of urease production and biofilm formation. These features enable them to contribute to both infection persistence and stone matrix formation, particularly in catheter-associated or recurrent infections [73].

Although *Escherichia coli* does not produce urease, it has been shown to promote calcium oxalate crystal growth, likely through surface interactions and reduction in urinary citrate. Additionally, *Escherichia coli* forms robust biofilms, enhancing its ability to persist in the urinary tract and contribute to stone-related infections [87].

## 8. Antibiotics and Urinary Dysbiosis

The administration of antibiotics has a profound impact on the urinary microbiome, often resulting in dysbiosis—a disruption of the native microbial equilibrium. While antimicrobial therapy remains indispensable for the management of urinary tract infections (UTIs), especially those caused by uropathogens like *Escherichia coli* and *Proteus mirabilis*, accumulating evidence indicates that broad-spectrum antibiotics can indiscriminately deplete both pathogenic and commensal bacterial taxa.

Agents such as fluoroquinolones (e.g., ciprofloxacin) and third-generation cephalosporins (e.g., ceftriaxone)—commonly used as first-line or empirical therapies—have been shown to significantly reduce microbial diversity, especially among protective genera like *Lactobacillus* and *Corynebacterium* [88]. This reduction not only impairs colonization resistance but also facilitates overgrowth of lithogenic and antibiotic-resistant organisms such as *Klebsiella pneumoniae* and *Enterococcus faecalis* [89].

Moreover, repeated or prolonged exposure to antibiotics such as trimethoprim–sulfamethoxazole or β-lactam combinations has been associated with persistent alterations in urinary microbiota and impaired recovery of oxalate-degrading species like *Oxalobacter formigenes*, potentially contributing to calcium oxalate stone formation [90]. In contrast, narrow-spectrum agents like fosfomycin and nitrofurantoin appear to exert a milder ecological footprint, preserving key microbial communities when used appropriately [91]. These findings emphasize the importance of drug selection and stewardship, particularly in recurrent UTI management, where preserving microbial homeostasis is critical to minimizing long-term complications such as stone recurrence and chronic inflammation [92].

Long-term antibiotic use also modifies the urinary microbiome, contributing to dysbiosis and loss of protective oxalate-degrading bacteria such as *Oxalobacter formigenes* [22,93]. In addition to microbial depletion, prolonged or broad-spectrum antibiotic exposure selects for antimicrobial resistance (AMR) genes within the urinary microbiome—many of which are carried on mobile genetic elements such as plasmids. A recent metagenomic study identified over 600 high-confidence plasmid sequences from urinary bacterial isolates, commonly harboring AMR genes and virulence factors in *Escherichia coli* and *Enterococcus faecalis* [94].

Within biofilm-associated communities—common in stone-forming conditions—horizontal gene transfer via conjugation is enhanced, facilitating rapid dissemination of resistance [94]. These plasmid-mediated mechanisms not only complicate antibiotic therapy by promoting treatment-resistant uropathogens but may also indirectly support lithogenesis through persistent colonization with urease-producing or biofilm-forming organisms. Clinically, this shift necessitates updated antibiotic stewardship and microbiological monitoring; detection of urinary plasmid-encoded AMR may guide more targeted treatment decisions and prompt use of narrow-spectrum agents, adjunctive microbial therapies, or phage-based strategies to disrupt resistant biofilms [95].

### 8.1. Mechanisms of Microbiome Alteration

Antibiotics do not distinguish between pathogens and commensal bacteria, resulting in the following conditions:

1. Loss of microbial diversity and a decline in protective taxa such as *Lactobacillus*, allowing opportunistic species like *Enterobacteriaceae* to proliferate.
2. Selective depletion of key organisms involved in urinary tract homeostasis.
3. Expansion of resistant strains and antibiotic-tolerant populations [35].

### 8.2. Antibiotic Class and Duration: Transient vs. Lasting Effects

Broad-spectrum antibiotics such as fluoroquinolones and beta-lactams are associated with long-term shifts in the urinary microbiome, whereas agents like nitrofurantoin and fosfomycin may have more transient effects [85]. Duration and frequency of exposure are also critical—longer or repeated courses increase the likelihood of persistent dysbiosis.

Microbial restoration following antibiotic exposure can take several months, and in some cases, the original microbial composition may never fully recover [92].

### 8.3. Clinical Evidence of Antibiotic-Induced Dysbiosis

Several human studies have documented urinary dysbiosis following antibiotic use:

1. Patients with a history of urolithiasis often exhibit microbiome alterations post-treatment, characterized by reduced *Lactobacillus* and increased *Proteus*, *Klebsiella*, or *Enterococcus* species [96].
2. Kidney transplant recipients receiving prophylactic antibiotics demonstrate significant loss of diversity and expansion of potentially resistant uropathogens [96].
3. Metagenomic analyses reveal that microbial disruptions may persist up to six months post-therapy, with downstream consequences for urinary health and microbial resilience [67].

These findings highlight the importance of considering not only antimicrobial efficacy but also the ecological consequences of treatment. Personalized antibiotic selection, informed by urinary microbiome profiling, may represent a key advance in microbiome-aware clinical practice [97].

## 9. Precision Approaches in Managing Urinary Microbiome Imbalances

The integration of urinary microbiome profiling into clinical care marks a turning point in how we approach urological disorders such as urolithiasis. Traditional therapeutic regimens often ignore the microbiota’s complexity, potentially undermining treatment outcomes. Emerging evidence supports a precision medicine framework, wherein diagnostics and treatments are customized to the individual’s urinary microbial composition [54,83].

For example, the identification of distinct microbial signatures in stone formers—such as the predominance of *Enterobacteriaceae*, *Proteus*, or depletion of *Lactobacillus*—enables individual risk stratification and targeted intervention. By leveraging next-generation sequencing (NGS), clinicians can predict lithogenic risk and tailor interventions to promote microbial homeostasis [23,67].

Moreover, precision tools such as microbial metabolic modeling offer the ability to simulate the ecological effects of specific antimicrobials or probiotic supplementation, further enhancing therapeutic precision [88]. Integration of urinary microbiome data with imaging, metabolomics, and host immune profiling may also identify patient subgroups who favorably respond to specific agents, reducing adverse effects and recurrence [11].

This personalized approach may also reduce unnecessary exposure to broad-spectrum antibiotics, thereby minimizing antimicrobial resistance and collateral damage to beneficial urobiota. Future clinical trials incorporating urinary microbiome stratification will be essential to validate the clinical utility of this emerging paradigm.

## 10. Microbiome-Targeted Therapies: Probiotics, Prebiotics, and Bacteriotherapy

Therapeutic manipulation of the urinary microbiome is increasingly recognized as a viable strategy to prevent or manage urological conditions, particularly urolithiasis and recurrent UTIs. Unlike antibiotics, microbiome-targeted therapies aim to restore ecological balance without inducing dysbiosis or resistance [92].

### 10.1. Action Mechanism of Probiotics and Prebiotics

Probiotics, such as *Lactobacillus crispatus* and *Lactobacillus rhamnosus*, have demonstrated multifaceted mechanisms that contribute to urinary tract health. These include competitive exclusion of uropathogens via mucosal adhesion, secretion of antimicrobial compounds such as lactic acid and hydrogen peroxide, and disruption of biofilms that shield pathogenic bacteria [35]. Additionally, probiotics modulate host immunity by enhancing secretory IgA production and promoting regulatory T-cell activity, thereby reducing inflammation-mediated epithelial damage. From a biochemical perspective, probiotic-induced acidification of urine creates an unfavorable environment for stone-forming ions, while certain strains can also support oxalate degradation directly or via synergistic action with oxalotrophic bacteria [81].

Prebiotics—non-digestible fermentable fibers such as inulin or galacto-oligosaccharides—act by selectively stimulating the growth of beneficial microbial taxa, notably *Bifidobacterium* and *Lactobacillus*. Their fermentation in the colon produces short-chain fatty acids (SCFAs), such as butyrate, which reinforce gut barrier integrity and modulate systemic inflammation. These metabolic effects can indirectly influence urinary metabolite profiles, particularly by reducing urinary calcium and oxalate excretion. Prebiotic-driven enrichment of oxalate-degrading species like *Oxalobacter formigenes* may further attenuate enteric oxalate absorption, a key pathway in calcium oxalate stone formation [98].

Another promising avenue is bacteriotherapy, including live biotherapeutic products (LBPs) and vaginal microbiota transplants (VMTs), which aim to reintroduce or rebalance functional microbial communities in the urogenital tract [99,100]. Such interventions may be tailored to address specific microbial deficiencies, similar to protocols used in fecal microbiota transplantation.

Moreover, these strategies may serve as adjunctive therapies during antibiotic treatment, supporting microbiota resilience and post-treatment recovery. As the field evolves, regulatory frameworks and microbial safety profiling will be critical to ensure consistent and effective outcomes in microbiome-targeted interventions.

### 10.2. Clinical Implications and Recommendations

The translation of microbiome research into therapeutic strategies for urolithiasis remains a key challenge. While observational studies and small-scale trials suggest the potential utility of probiotics, prebiotics, and microbiome-conscious antibiotic selection, robust clinical guidelines are still lacking. Nevertheless, early evidence supports the integration of microbiome modulation into urological practice, especially for high-risk patients with recurrent stone disease or frequent antibiotic exposure [98].

Probiotic strains such as *Lactobacillus plantarum*, *Lactobacillus casei*, and *Oxalobacter formigenes* have been shown to degrade oxalate, reduce urinary pH, inhibit biofilm formation, and modulate local inflammation—mechanisms relevant to both infection-induced and metabolically induced lithogenesis [51]. These functional effects may be particularly beneficial in patients who have experienced antibiotic-induced depletion of protective taxa. Prebiotics, including inulin and fructooligosaccharides, further support the proliferation of beneficial microbial communities by providing fermentable substrates that yield short-chain fatty acids (SCFAs). SCFAs can enhance mucosal barrier integrity, reduce intestinal oxalate absorption, and modulate systemic inflammation, thereby influencing the gut–kidney axis in lithogenic processes [98].

However, the clinical implementation of these interventions is complicated by variability in microbial colonization, product standardization, and host-specific responses. Additionally, the lack of regulatory oversight in many probiotic formulations raises concerns about strain viability, dosage consistency, and long-term safety [101]. Therefore, while these agents hold promise as adjuncts to conventional therapies, their use should currently be guided by risk stratification—targeting patients with recurrent stones, a history of antibiotic use, or identifiable microbiota dysbiosis [51,92].

Importantly, antibiotic stewardship tailored to microbiome preservation must also be considered. Preferential use of narrow-spectrum agents and avoidance of unnecessary broad-spectrum antibiotics may help reduce collateral damage to commensal urinary flora. Concurrent or sequential use of validated probiotic formulations during and after antibiotic regimens may further support microbial recovery and reduce stone recurrence risk. Future directions should include well-powered clinical trials to assess efficacy, optimal dosing, and strain-specific effects, as well as development of pharmacobiotic guidelines specific to stone formers [96].

## 11. Discussion

Recent advances in urinary microbiome research have emphasized the urinary tract as a non-sterile, dynamically colonized environment. In alignment with the emerging literature, our review underscores the dual role of microbial and metabolic factors in urolithiasis pathogenesis. Specifically, antibiotic therapies—although clinically necessary—can disrupt microbial equilibrium and increase susceptibility to recurrent stone formation. This is consistent with findings that recurrent antibiotic exposure facilitates the expansion of lithogenic pathogens such as *Proteus mirabilis* and *Klebsiella pneumoniae* while depleting protective species like *Lactobacillus* and *Oxalobacter formigenes* [2].

The pathogenic mechanisms identified—urease activity, citrate degradation, and biofilm formation—are well established in the literature as contributing to stone formation through local alkalinization, crystal nucleation, and immune evasion. Our synthesis reinforces the concept that bacterial biofilms not only provide resistance to antimicrobial agents but also act as structural scaffolds for mineral deposition and stone persistence within the urinary tract [12,72].

Moreover, antibiotic-induced dysbiosis has been shown to impair colonization by oxalate-degrading species such as *Oxalobacter formigenes*, which normally mitigate calcium oxalate supersaturation by reducing intestinal oxalate absorption [35]. Disruption of these microbial populations may, therefore, directly contribute to hyperoxaluria and enhanced lithogenic potential [90].

Comparative analyses of microbiota in stone-formers across studies consistently reveal a shift toward *Enterobacteriaceae* dominance, reduced microbial richness, and ecological instability [60,71]. These findings are further supported by metagenomic data indicating long-lasting microbiome alterations even months after antibiotic therapy [67].

Importantly, antibiotics differ in their degree of microbiota disruption and selective pressure on the urinary microbial community. Narrow-spectrum agents such as fosfomycin and nitrofurantoin have been associated with more transient microbiota changes and may be preferable in recurrent urinary tract infection (UTI) management where microbial preservation is critical [42].

Nonetheless, several limitations persist across studies, including small sample sizes, inconsistent methodology, and insufficient control for confounders such as prior antibiotic exposure. Additionally, while 16S rRNA sequencing provides valuable taxonomic information, it lacks the functional resolution needed to distinguish commensals from pathogens or assess metabolic contributions to disease [6]. Integrating multi-omic approaches—including metabolomics and transcriptomics—alongside longitudinal sampling will be essential for capturing dynamic host–microbiome interactions over time [82].

Emerging microbiome-preserving interventions—including targeted probiotics, metabolic modulators, and microbiota-informed antibiotic stewardship—present promising therapeutic avenues. However, clinical validation through robust, controlled trials remains essential to establish their efficacy in preventing recurrence and preserving urinary tract health [51,64].

## 12. Conclusions

Urolithiasis is increasingly recognized as a multifactorial disorder in which the urinary microbiome plays a pivotal role alongside metabolic, dietary, genetic, and systemic factors. Disruptions to microbial homeostasis—particularly those induced by antibiotics—can facilitate lithogenesis through mechanisms including urine alkalinization, inflammation, and microbial metabolite alterations. Antibiotic-induced dysbiosis not only reduces beneficial taxa such as *Lactobacillus* and *Oxalobacter formigenes* but also promotes colonization by urease-producing, biofilm-forming pathogens, thereby complicating prevention strategies and increasing recurrence risk. This underscores the urgent need for microbiome-conscious stewardship in urological care. As the microbiota emerges as both a contributor to and potential therapeutic target in stone disease, novel interventions—such as targeted probiotics, precision antimicrobials, and dietary modulation—offer promising avenues for individualized prevention. Integration of microbial profiling with host metabolic and immune parameters may enable precision medicine approaches that optimize both efficacy and long-term outcomes. Continued investment in longitudinal, multi-omic research is essential to advance microbiome-informed management strategies and reduce the global burden of kidney stone disease.

## Figures and Tables

**Table 1:** Sex-related differences in urolithiasis.

Sex/Gender	Stone Incidence	Key Features	Microbiota Influence
Male	Historically higher	Higher urinary excretion of calcium oxalate and other solutes	Less diverse urinary microbiota; higher risk profile
Female (premenopausal)	Lower than men	Estrogen increases urinary citrate and modulates microbiota diversity	Protective microbial diversity linked to hormonal modulation
Female (postmenopausal)	Gap narrowing	Loss of estrogen may reduce protective effects	Reduced diversity; convergence with male profiles

**Table 2:** Role of obesity in renal infections and microbiome modulation.

Obesity-Related Factors	Effect on Renal Health
Low urinary pH and uric acid supersaturation	Promotes uric acid stone formation and inhibits solute dissolution
Hyperoxaluria and increased stone-forming solutes	Favors calcium oxalate crystallization
Chronic low-grade inflammation	Impairs mucosal immunity and increases infection risk
Altered gut and urinary microbiome	Reduces microbial diversity; promotes dysbiosis
Increased colonization by uropathogens	Enhances biofilm formation and recurrent UTIs

**Table 3:** 16S rRNA sequencing vs. conventional culture-based method.

Feature	16S rRNA Sequencing	Conventional Culture
Viability requirement	Detects both viable and non-viable bacteria	Detects only viable bacteria
Detection of low-abundance species	High sensitivity; detects low-abundance species	Low sensitivity; may miss rare species
Culture dependency	Culture-independent	Requires specific culture media
Taxonomic resolution	High; provides detailed bacterial identification	Limited; may identify only genus or group
Functional/metabolic information	Not provided	Partially, through observable growth and behavior
PCR bias	Possible introduction of amplification bias	Not applicable
Time to results	Short (depending on platform and analysis pipeline)	Longer (due to incubation periods)

**Table 4:** Comparative table: urolithogenic mechanism of key bacterial species.

Bacterial Species	Urease Production	Urine Alkalinization	Biofilm Formation	Citrate Lyase Activity	Crystal Growth/Aggregation	Associated Stone Type (s)
*Proteus* spp.	high	yes	crystalline biofilm	not reported	yes	struvite, apatite
*Klebsiella* spp.	moderate	yes	yes	not reported	yes	struvite, calcium oxalate
*Escherichia coli*	no	no	yes	yes	yes	calcium oxalate, struvite (via biofilm)

## Data Availability

Not applicable.
